# Transjugular Tricuspid Edge-to-Edge Repair for Hostile Inferior Vena Cava Anatomy

**DOI:** 10.1016/j.jaccas.2026.109420

**Published:** 2026-08-04

**Authors:** Gioel Gabrio Secco, Edoardo Elia, Chiara Sartori, Giulia Maj, Marco Lombardi, Andrea Audo, Giuseppe Patti

**Affiliations:** a Division of Cardiology, University Hospital SS Antonio e Biagio e Cesare Arrigo, Alessandria, Italy; b Department of Translational Medicine, University of Eastern Piedmont, Novara, Italy; c Division of Cardiac Intensive Care Unit, University Hospital SS Antonio e Biagio e Cesare Arrigo, Alessandria, Italy; d Division of Cardiac Surgery, University Hospital SS Antonio e Biagio e Cesare Arrigo, Alessandria, Italy; e Division of Cardiology, University Hospital Maggiore della Carità, Novara, Italy

**Keywords:** jugular access, tricuspid regurgitation, tricuspid transcatheter edge-to-edge repair

## Abstract

**Objective:**

To describe a step-by-step transjugular approach for tricuspid transcatheter edge-to-edge repair (T-TEER) when hostile inferior vena cava anatomy precludes safe transfemoral access.

**Key Steps:**

We describe a case transjugular T-TEER in a patient with severe tricuspid regurgitation and prohibitive inferior vena cava anatomy. Distal right jugular access was obtained under ultrasound guidance with preclosure. Progressive dilation allowed advancement of the guiding catheter over stiff guidewire. Navigation toward tricuspid valve required awareness of reversed catheter movements to maintain orientation. Two devices were implanted in the anteroseptal position under echocardiographic guidance, using the first clip as landmark for the second one. The system was straightened before withdrawal.

**Potential Pitfalls:**

Large-bore jugular access may increase risk of vascular complications; distal puncture and gradual dilation are essential. Reversed catheter movements may cause disorientation or interaction with atrial structures.

**Take-Home Message:**

Transjugular access represents a feasible alternative for T-TEER when transfemoral delivery is unsafe.

## Visual Summary

**Figure:**
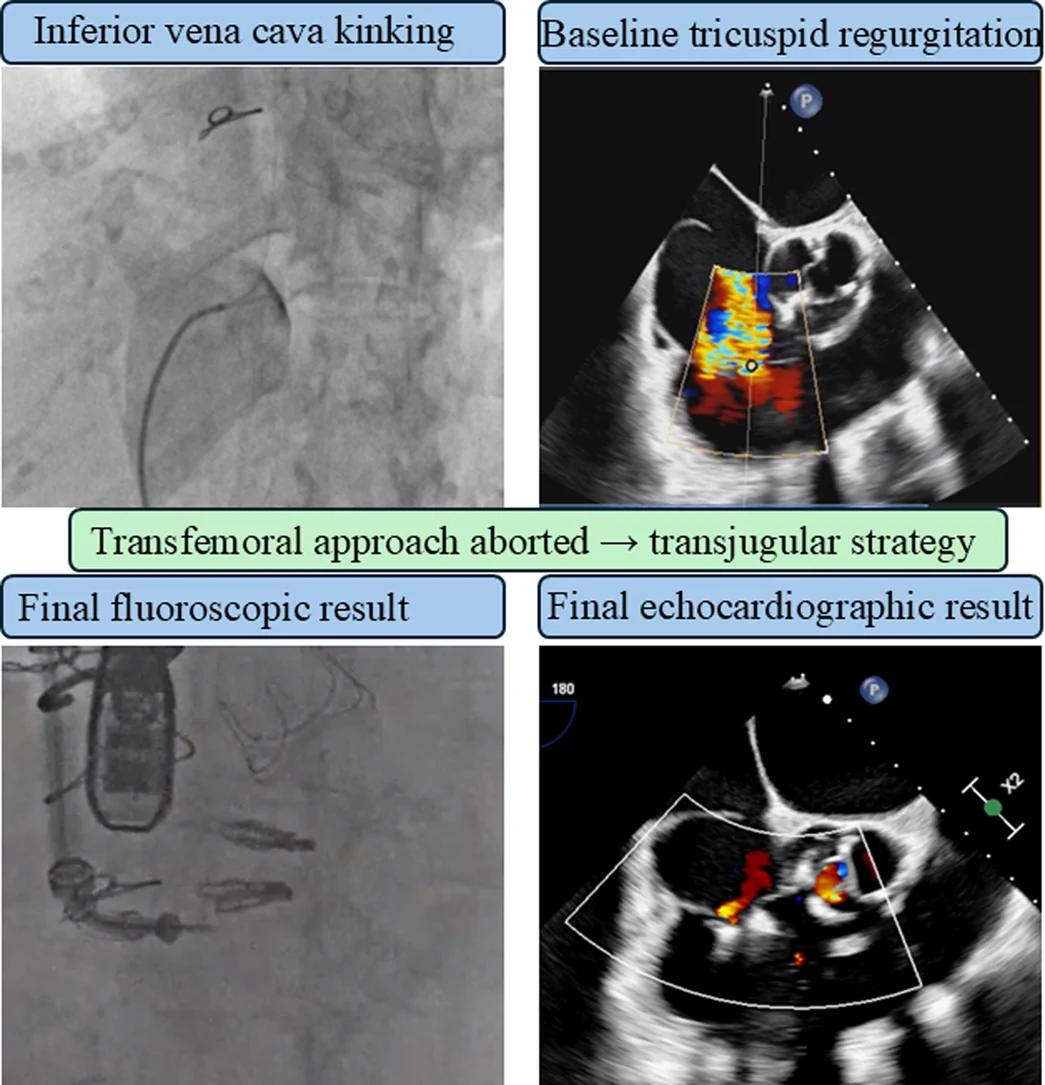

**Visual Summary:**
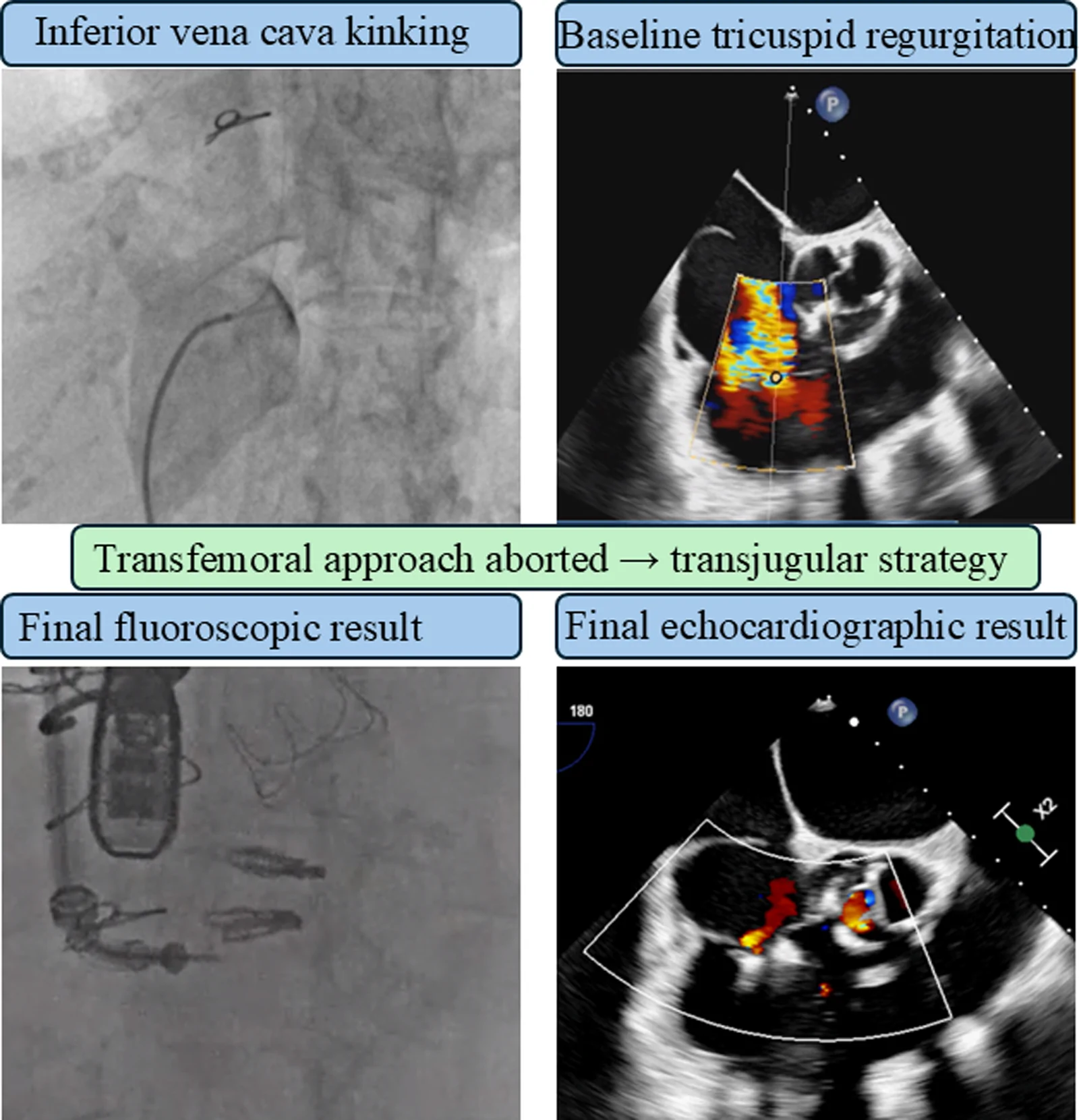
Transjugular Tricuspid Transcatheter Edge-to-Edge Repair for Hostile Inferior Vena Cava Anatomy

Severe tricuspid regurgitation (TR) is increasingly recognized as a major contributor to reduced exercise capacity and heart failure hospitalization, with consequent poor functional status and clinical outcomes, especially in patients at high surgical risk. Tricuspid transcatheter edge-to-edge repair (T-TEER) has become an effective therapeutic option to reduce TR severity and improve symptoms in selected patients.^1^, ^2^, ^3^ The procedure is usually performed via transfemoral venous access, which provides a favorable trajectory for large-bore delivery systems. However, marked inferior vena cava (IVC) tortuosity or kinking may render this route unsafe by increasing the risk of vascular injury when introducing a 25-F guide.^1^, ^2^, ^3^, ^4^ In this report, we describe a step-by-step transjugular approach for T-TEER as an alternative access strategy in the setting of hostile IVC anatomy, highlighting key technical maneuvers to ensure procedural safety and reproducibility.

**Take-Home Messages**

• Hostile inferior vena cava anatomy may represent an underrecognized limitation for transfemoral tricuspid edge-to-edge repair (T-TEER).
• Transjugular T-TEER can safely expand treatment options in selected patients, provided operators adapt to reversed catheter movements and maintain spatial orientation.

## Case Summary

A 74-year-old woman with recurrent right-sided heart failure symptoms (NYHA functional class III) was referred for treatment of severe to torrential functional TR. Her medical history included coronary artery bypass grafting (left internal mammary artery to the left anterior descending artery) and surgical aortic valve replacement with a 23-mm bioprosthesis. Echocardiography showed preserved biventricular systolic function, tricuspid annular dilation, and an 8-mm coaptation gap between the septal and anterior leaflets (Figures 1A and 1C, Videos 1 and 2). After heart team discussion, T-TEER was selected given the patient's elevated surgical risk (Society of Thoracic Surgeons predicted risk of mortality: 4.26% and composite morbidity and mortality: 20.9%). A transfemoral attempt was discontinued because marked IVC angulation and tortuosity prevented safe advancement of the 25-F guiding system, as confirmed by computed tomography. The procedure was therefore completed via a transjugular approach. Two clips were implanted in the anteroseptal position under echocardiographic and fluoroscopic guidance, resulting in mild residual TR without a significant transvalvular gradient. Jugular hemostasis was achieved without access-site complications, and the patient was discharged with early clinical improvement.

**Figure 1:**
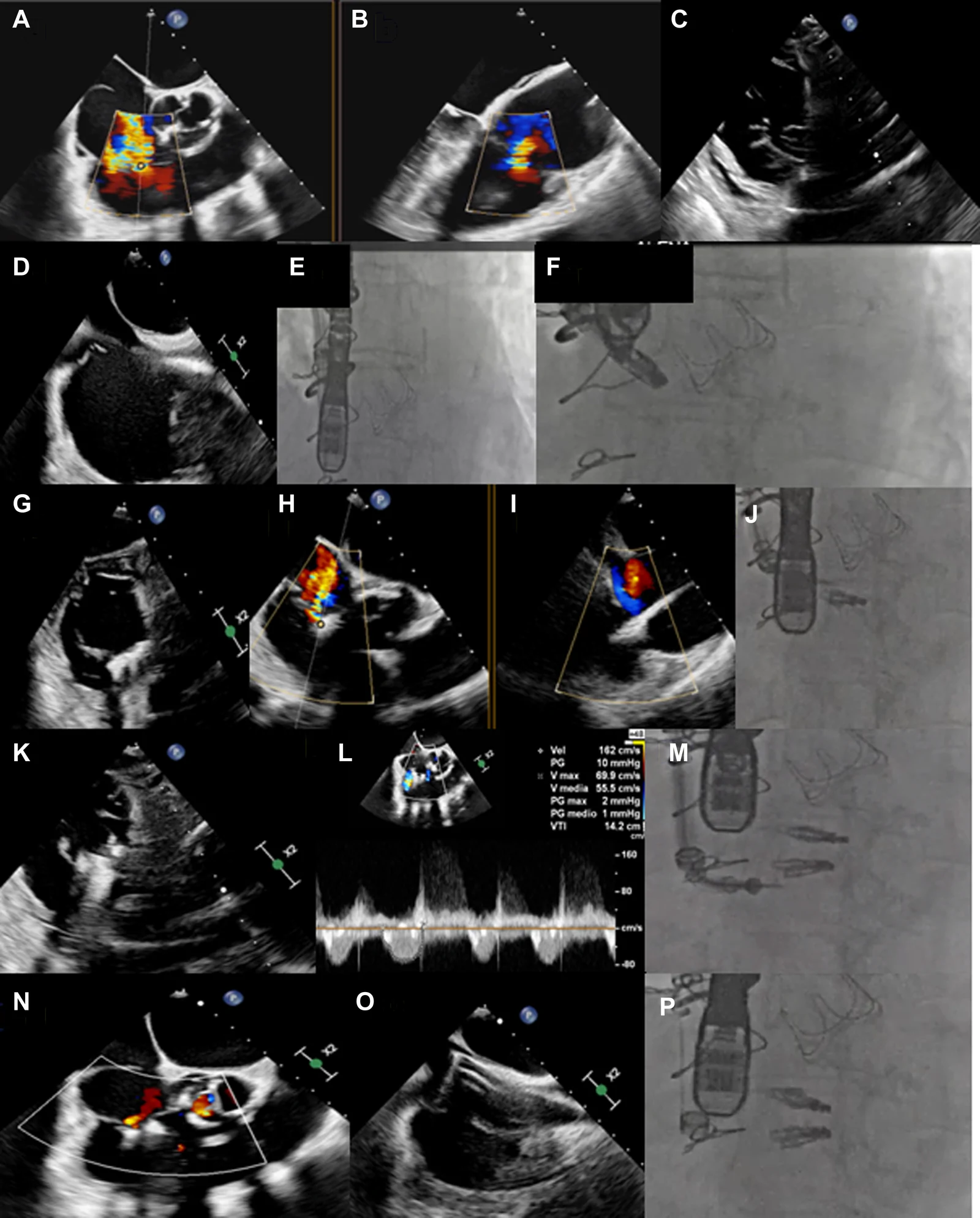
Transjugular Transcatheter Tricuspid Edge-to-Edge Repair (A and B) baseline tricuspid regurgitation; (C) 8-mm coaptation gap; (D and E) straightened guiding catheter in right atrium; (F) flex toward tricuspid valve; (G) first device orientation; (H to J) result after first device implant; (K) second device orientation; (L) mean gradient after second device implant; (M and N) final result after second device implant; (O and P) straightened empty guiding catheter in right atrium before removal.

## Procedural Steps

All procedural equipment is summarized in Table 1.

**Table 1:** Equipment List for Transjugular Tricuspid Transcatheter Edge-to-Edge Repair

Equipment	Size/Type	Purpose
Venous access sheath	Short 6-F introducer sheath	Initial distal right internal jugular venous access under ultrasound guidance
Ultrasound probe	Linear probe and transesophageal probe	Ultrasound-guided venous puncture and procedural transesophageal guidance
Guidewire (stiff)	0.035-inch stiff guidewire	Support for progressive dilation and large-bore system advancement
Pre-closure device	ProGlide (Abbott Vascular)	Preclosure before upsizing to facilitate final hemostasis
Dilators	Progressive dilator set	Stepwise dilation prior to 25-F system insertion
T-TEER device	TriClip G4 system (XTW) (Abbott)	Tricuspid edge-to-edge repair; 2 devices implanted in this case

T-TEER = tricuspid transcatheter edge-to-edge repair.

### Step 1. Preprocedural planning, transfemoral setup, and recognition of hostile IVC anatomy

TR mechanism and suitability for T-TEER on transthoracic and transesophageal echocardiography (leaflet anatomy, coaptation gap, and target jet) were confirmed before heart team discussion. T-TEER was initially planned using the standard transfemoral approach, right femoral venous access was obtained with ultrasound-guided puncture, and the IVC trajectory was assessed under fluoroscopy during wire advancement. Severe IVC kinking and angulation were encountered, and the vessel could be negotiated only with a coronary guidewire and a coronary diagnostic catheter. Therefore, given the high risk of vascular injury or IVC rupture with large-bore device advancement, the transfemoral strategy was discontinued before introducing the 25-F guiding catheter. The venous anatomy was then reassessed by reviewing available data, and computed tomography confirmed that transfemoral delivery was unsafe.

### Step 2. Catheterization laboratory preparation and procedural setup

The catheterization laboratory configuration is illustrated in Figure 2. The procedure was performed under general anesthesia with continuous transesophageal echocardiographic guidance. The primary and secondary operators stood on the patient's right side near the head, with the table positioned above the access site to allow a stable and coaxial position of the system. On the patient's left side were the anesthesia team and the cardiac imaging specialist performing transesophageal echocardiography, with the anesthesiologist positioned slightly more cranially than the imaging specialist.

**Figure 2:**
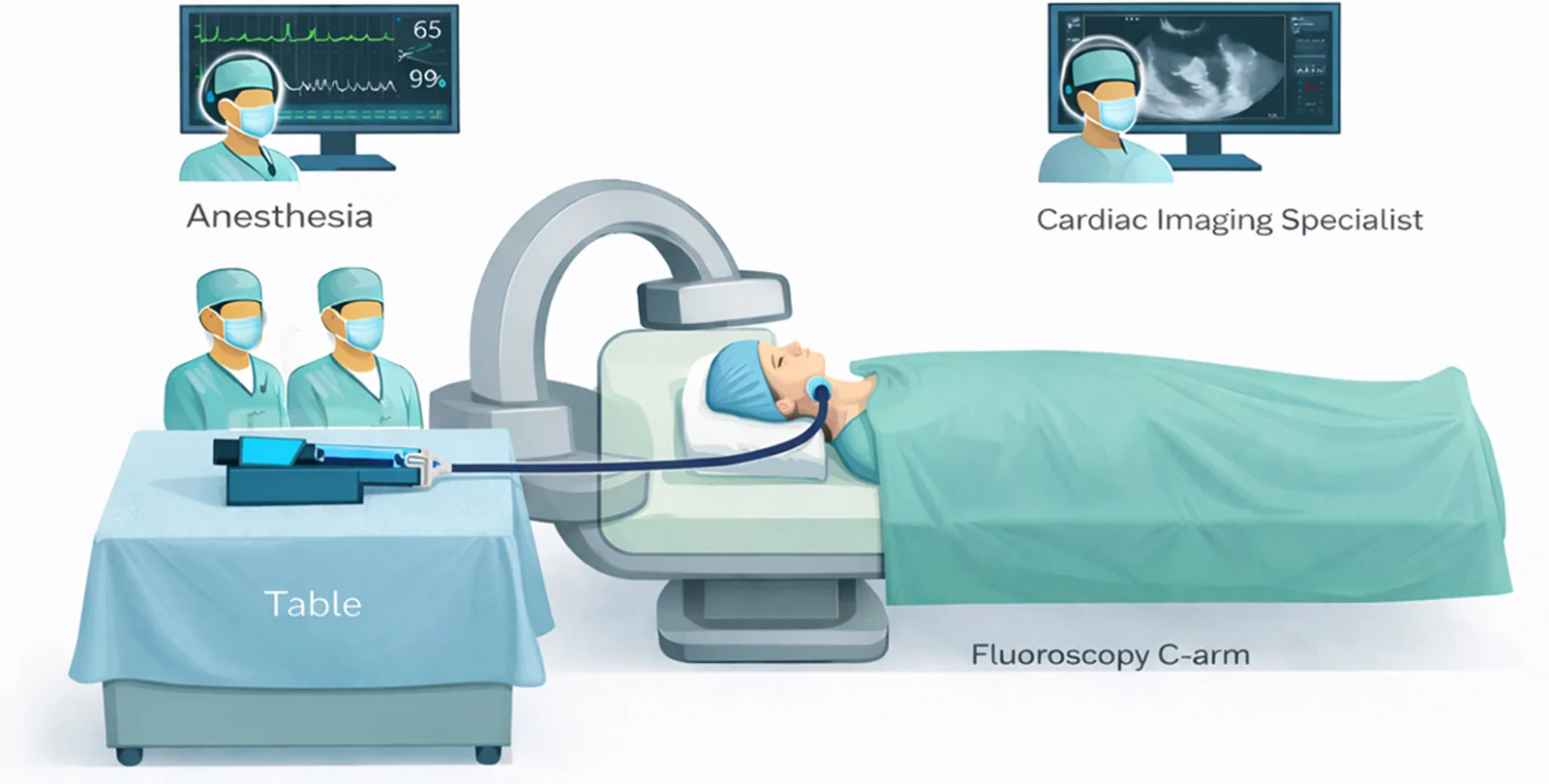
Catheterization Laboratory Setup for Transjugular Transcatheter Tricuspid Edge-to-Edge Repair

### Step 3. Transjugular access site selection, imaging guidance, and preclosure

Distal right internal jugular venous access was obtained under ultrasound guidance with a 6-F introducer sheath, intentionally avoiding the jugular-subclavian junction to reduce the risk of vascular injury. A single suture-based closure device was deployed before upsizing to facilitate final hemostasis.

### Step 4. Dilation and advancement of the 25-F guiding system

Progressive dilation was performed over a stiff 0.035-inch guidewire. A straightened 25-F guiding catheter was advanced under fluoroscopy with liberal lubrication (saline and propofol were used in this case) to improve deliverability and reduce venous trauma (Figures 1D and 1E, Video 3).

### Step 5. Right atrial navigation and device implantation with reversed catheter mechanics

Compared with the standard transfemoral approach, the transjugular trajectory results in an inverted spatial orientation of the delivery system. When entering the right atrium from the superior vena cava, the guiding catheter approaches the tricuspid valve from a cranial direction rather than from the IVC. As a consequence, several catheter maneuvers differ from those used in the standard transfemoral technique.

First, forward advancement toward the tricuspid valve is achieved by controlled catheter pullback, rather than by pushing the system forward as in the transfemoral approach once the device has been properly prepared and oriented. This requires gradual and deliberate movements to maintain stability and avoid interaction with right atrial structures.

Second, rotational maneuvers appear reversed, and clockwise or counterclockwise rotation may generate opposite spatial responses compared with the femoral trajectory. Similarly, movements of the delivery catheter knobs are inverted (both S/L and +/−). Continuous echocardiographic guidance is therefore essential to maintain and continuously check spatial orientation and avoid interaction with anatomical structures such as the Eustachian valve or Chiari network.

Third, achieving coaxial alignment with the tricuspid valve plane may be more challenging from the superior approach. In this context, it is important to note that only the delivery catheter maneuvers are reversed, whereas device movements remain standard, including those controlled by the flex/extension knob when the delivery catheter is correctly oriented. Coaxial alignment was facilitated by gradual flexion of the guiding catheter combined with subtle rotational adjustments under simultaneous fluoroscopic and transesophageal echocardiographic guidance.

In our case, flexion with the flex knob combined with controlled pullback allowed movement from a lateral to a septal orientation toward the tricuspid valve while avoiding interaction with a prominent Eustachian valve (Figure 1F, Video 4). Once adequate alignment was achieved, the first device was advanced and oriented in the transgastric view (Figure 1G, Video 5), and subsequently implanted in the anteroseptal commissural region targeting the dominant regurgitant jet (Figures 1H to 1J, Videos 6 and 7). After evaluation of the initial result, a second device was positioned adjacent to the first, using the first clip as a fluoroscopic landmark in combination with the transgastric view to optimize positioning (Figure 1K). This strategy improved leaflet coaptation and resulted in effective reduction of TR without a significant increase in the mean gradient (Figures 1L and 1N, Video 8). The guiding catheter was then removed straightened and empty (Figures 1O and 1P, Video 9). The main technical differences between transfemoral and transjugular approaches are summarized in Table 2.

**Table 2:** Key Differences Between Transfemoral and Transjugular Tricuspid Transcatheter Edge-to-Edge Repair Approaches

Aspect	Transfemoral Approach	Transjugular Approach
Venous access route	Inferior vena cava	Superior vena cava
Catheter movements (push/pull)	Forward advancement moves the system toward the aorta and laterally. Conversely, pullback the system moves it posteriorly	The effects of the movements are inverted: Forward advancement moves the system posteriorly. Conversely, pullback the system moves it toward the aorta and laterally
Rotational response	Clockwise rotation directs the system septally; counterclockwise rotation directs it laterally	Rotational responses are inverted: Counterclockwise rotation directs the system septally, whereas clockwise rotation directs it laterally
+/− knob of delivery catheter	Increasing (+) or decreasing (−) curvature modifies catheter height	Knob response is inverted, resulting in opposite changes in catheter height
S/L knob of delivery catheter	Move the device septally (S) or laterally (L)	Knob response is inverted, move the device septally (L) or laterally (S)
F/E knob of clip handle	Flex (F) and extend (E) the distal part of the system	Same behavior as in the transfemoral approach

### Step 6. Final assessment and safe system withdrawal

Residual TR and the mean transtricuspid gradient were assessed to confirm an effective result without clinically relevant stenosis (Figures 1L to N). After deployment of the second device, the guiding catheter was fully straightened before withdrawal (Figures 1O and 1P), and hemostasis was achieved with the preclosure sutures without access-site complications.

## Potential Pitfalls

A key challenge is the early recognition of hostile IVC anatomy. Severe kinking or angulation may still allow passage of diagnostic catheters or guidewires but can make transfemoral advancement of a large-bore 25-F guiding system unsafe; therefore, prompt reassessment of venous anatomy using available imaging is crucial. A second critical aspect is right internal jugular access management. Proximal puncture close to the jugular-subclavian junction and inadequate closure planning may increase the risk of bleeding or vascular injury; distal ultrasound-guided puncture, preclosure before upsizing, and careful confirmation of hemostasis after withdrawal are essential. Finally, operators must anticipate loss of orientation related to inverted catheter mechanics with the transjugular approach. Small controlled maneuvers under continuous echocardiographic and fluoroscopic guidance help maintain spatial awareness and reduce interaction with right atrial structures.

Another relevant consideration concerns leaflet accessibility according to the access route. With the transjugular approach, the superior trajectory may facilitate access to the septal and anterior leaflets, as well as the anteroseptal commissure, whereas more lateral or posterior targets may require additional catheter manipulation to achieve optimal orientation. In the present case, the anteroseptal commissure was readily accessible, although precise positioning required careful adjustments to maintain coaxial alignment. These observations suggest that grasping strategies should be adapted according to the direction of approach and individual valve anatomy.

## Conclusions

Transjugular access represents a feasible alternative for tricuspid transcatheter edge-to-edge repair when transfemoral delivery is limited by hostile inferior vena cava anatomy. Careful preprocedural assessment of venous anatomy and awareness of inverted catheter mechanics are essential to ensure procedural safety and effective reduction of tricuspid regurgitation.

## Funding Support and Author Disclosures

The authors have reported that they have no relationships relevant to the contents of this paper to disclose.

## Appendix

Video 1 Baseline Tricuspid Regurgitation

Video 2 Coaptation Gap

Video 3 Guiding Catheter in Right Atrium

Video 4 : Flex Toward Tricuspid Valve

Video 5 Orientation of First Device

Video 6 Grasping and Result After First Device

Video 7 Flex of Second Device

Video 8 Second Grasping and Final Outcome

Video 9 Empty Guiding Catheter in Right Atrium Before Removal
